# Comparing serum levels of cardiac biomarkers in cancer patients receiving chemotherapy and subjects with chronic periodontitis

**DOI:** 10.1186/1479-5876-10-S1-S5

**Published:** 2012-09-19

**Authors:** Wings TY Loo, Yuan Yue, Chang-bin Fan, Lan-jun Bai, Yi-ding Dou, Min Wang, Hao Liang, Mary NB Cheung, Louis WC Chow, Jin-le Li, Ye Tian, Liu Qing

**Affiliations:** 1 UNIMED Medical Institute, Hong Kong SAR; 2 School of Chinese Medicine, Li Ka Shing Faculty of Medicine, The University of Hong Kong, Hong Kong SAR; 3 State Key Laboratory for Oral Diseases and Department of Prosthodontics, West China Hospital of Stomatology, Sichuan University, Sichuan, PR China; 4 Stomatological Hospital of Guangzhou Medical College, Guangzhou, PR China; 5 Department of Stomatology, Sichuan Academy of Medical Sciences & Sichuan Provincial People’s Hospital, Chengdu, Sichuan Province, P.R. China; 6 Jin Hua Dentistry, Chengdu, 610041, Sichuan Province, P.R. China; 7 Keenlink Dental Clinic, Hong Kong SAR

## Abstract

**Background:**

Chronic periodontitis (CP) is a chronic inflammation associated with elevations of several inflammatory and cardiac markers. Studies implicated CP as one of the etiologies in coronary heart disease (CHD). Cardiotoxicity is a major complication of anticancer drugs, including anthracyclines and 5-fluorouracil (5FU). The most severe cardiac complications are heart failure, arrhythmia and coronary heart disease (CHD). In this study, we compared the level of inflammatory factors and cardiac markers between chronic periodontitis patients and cancer patients receiving chemotherapy.

**Methods:**

108 blood samples of periodontally healthy subjects were obtained on random from Hong Kong Red Cross, and these represented the controlled population. Forty-four patients diagnosed with chronic periodontitis were recruited from the West China Hospital of Stomatology, Sichuan University. They have received scaling and root planning with mean pocket depths of 6.05 mm. Thirty breast cancer patients diagnosed with invasive ductal carcinoma from UNIMED Medical Institute, Hong Kong gave consent to participate in this study. They received 4 cycles of 500mg/m^2^ 5-fluorouracil, 75 mg/m^2^ epirubicin and 500mg/m^2^ cyclophosphamide at a 3-week interval between each cycle. Peripheral venous blood from each group was taken for measurement of blood cells, inflammatory marker (P-selectin, high sensitvity C-reactive protein) and cardiac markers (troponin T; troponin I; N-terminal pro brain natriuretic peptide (Nt-proBNP) and Lactate dehydrogenase (LDH).

**Results:**

The lymphocyte count was higher (p < 0.05) in periodontitis patients than the other two groups, and more neutrophils (p < 0.05) were seen in cancer patients receiving chemotherapy. The two test groups demonstrated higher levels (p < 0.01) of inflammatory and cardiac markers than the control group.

**Conclusions:**

The elevated cardiac markers found in periodontitis patients suggested that they may carry potential risks in developing cardiac lesions. Troponin T, troponin I, pro-BNP, LDH and high sensitvity C-reactive protein may be used as markers to monitor cardiac lesions in chronic inflammatory patients.

## Background

Gingivitis may lead to periodontitis without proper treatment; the inflammatory periodontal tissues will progress increasingly with age and may lead to pathological destruction of the tooth supporting tissues, tooth loosening and potential tooth loss [1,2]. The chronic inflammatory periodontal disease, chronic periodontitis (CP), is a common chronic infection with mainly gram-negative bacteria of the tissue surrounding the teeth. Its prevalence increases with increasing age [3]. Most of the adults in the US have periodontal disease, whereas a third of the elderly have moderate to severe periodontal disease [4]. Its common symptoms are bad breath, swollen or bleeding gums, and loose teeth, etc. A comprehensive examination is useful for diagnosing periodontitis and designing the treatment plan [5]. Common treatments include surgery, scaling and root planning and medication, however controlling the infection is an effective way of treating periodontitis. Recent studies have shown salivary compounds can be a good biomarker of periodontitis, such as proteins and immunoglobulins have been used and they showed satisfactory results [6,7].

Studies have also suggested a correlation between periodontitis and HIV [8,9]. In 1989 a study was carried out by Mattila and they found that dental health was strongly associated with myocardial infarction (MI) [10]. Others have shown that CP might be associated with stroke and diabetes [11,12]. due to the suppressed immune system. Many studies also suggested that CP was related to atherosclerosis coronary heart disease (CHD), which is one of the major causes of death in many developed countries [10,13-15]. A meta-analysis suggested a positive correlation (1.14-fold higher risk) between CP and CHD even after adjusting the risk factors [16]. Some studies also suggested that the risk of CHD may be lowered by periodontal treatments [17,18]. Therefore the relationship between CP and CHD has become highly controversial but difficult. The underlying causes of both CP and CHD are complicated because not only genetic factor plays a role, environmental factors are also involved. The traditional risk factors, such as age, sex, smoking, etc., cannot fully explain the causes. In recent decades, many studies have aimed at studying the association between chronic inflammation and CHD.

Cardiac markers are biomarkers which can evaluate the heart function by identifying the blood chemicals that are associated with MI. Lactate dehydrogenase (LDH) is an enzyme that is found in many different types of cells, especially the heart, kidney, liver and muscle. It is involved in energy production, which catalyses the conversion of lactate to pyruvate [19]. The LDH level contains the measurement of five different isoenzymes (LDH-1, LDH-2, LDH-3, LDH-4, and LDH-5) [20], and is released into the blood when the cell dies. Thus, its level can reflect the injury to cells, but it is not used to determine the damaged location. Troponins are proteins that are found in skeletal and cardiac muscle fibres. Troponin T and troponin I regulate the cardiac muscle contraction [21], they are only released into the blood when there is heart muscle damage. Therefore, they are specific to the heart muscle with a high sensitivity and specificity [22]. N-terminal pro b-type natriuretic peptide (NT pro-BNP) is most concentrated in the left ventricular myocardium. The protein pro-BNP volume remains low under normal conditions. It regulates the blood volume by cleaving itself to release BNP and NT-proBNP. During heart failure, however, the left ventricle works harder and produces more BNP and NT-proBNP [23]. There are high levels of BNP and NT-proBNP present in acute coronary syndromes and pulmonary thromboembolism [24].

Increased levels of chronic inflammation markers can be observed in CP patients [25,26]. The high sensitivity C-reactive protein (hs-CRP) level in periodontitis patients increases with the severity of the disease [27]. Hs-CRP became a novel risk factor for CHD and inflammation in recent years, and some studies have shown a strong association between hs-CRP expression level and increased risk of cardiovascular disease [28-34].

P-selectin is a transmembrane protein, which moves to the endothelial cell surface and recruits leukocytes to the inflammation site. Studies suggested that P-selectin deficiency causes less atherosclerotic lesions [35,36]; therefore, it has been proposed as a platelet activation marker in 1997 [37].

Some studies have suggested periodontitis may have an effect on oral, pancreatic, oesophageal and gastric cancer risk [38-42]. A significant association between breast cancer risk and periodontal disease was suggested by Hujoel [43]; but, Hiraki found no association between breast cancer risk and tooth loss [44]. Invasive ductal carcinoma (IDC) is the most common type of breast cancer. It can be treated by surgery, radiation therapy, anthracycline chemotherapy and hormonal therapy. Anthracyclines have proven to increase survival rates; however, it is accompanied by the adverse effect of cardiotoxicity. Signs of cardiovascular disease were observed in patients receiving fluorouracil [45]; cardiac damages were found in patients receiving cumulative dose of epirubicin [46]. Increased levels of troponin T were also found at the early anthracycline treatment stage [47].

The aim of this study was to evaluate the association of periodontal disease with CHD by examining the complete blood picture, selected inflammatory markers and cardiac biomarkers in subjects with moderate to severe chronic periodontitis, control subjects and cancer patients who have received chemotherapy.

## Methods

### Research subjects recruitment

108 blood samples of periodontally healthy subjects were recruited on random from Hong Kong Red Cross with their consent and these represented the control population. They underwent detailed oral examination at Keenlink Dental Clinic, Hong Kong. The subjects were determined to be free from the following: systemic or chronic diseases, smoking history, swelling of the lymph nodes, temporal mandibular joint disease, soft tissue abnormalities, severe dental caries, supragingival/subgingival calculus, furcation or generalized gingival recession. Full-mouth clinical parameters were recorded including plaque index, bleeding on probing, probing pocket depth and clinical attachment loss at six sites per teeth according to the American Academy of Periodontology in 1999 [48]. The mean pocket depth of these subjects was 2.71mm and their clinical attachment loss less than 1 mm in any one quadrant. None of the subjects were smokers in this study (Table 1).

**Table 1:** The clinical data (Mean ± SD) of control subjects and periodontitis patients

Clinical parameters	Control subjects N = 108	Periodontitis patients N = 44
Mean age	42.90(± 9.69)	49.27(±13.63)
Age range	18-60	18-74
Male/Female	69/39	26/18
Mean pocket depth (mm)	2.71(±1.23)	6.05(±2.74)*
Bleeding on probing (%)	40.26(±9.52)	78.15(±19.76)*
gingival recession (%)	1.0(±1.24)	38.92(±25.88)*
Supragingival calculus (%)	34.14(±13.62)	62.98(±25.84)

Significant different from the control, * *p* < 0.05

Forty-four patients diagnosed with chronic periodontitis were recruited from West China Hospital of Stomatology, Sichuan University. The diagnosis of chronic periodontitis was made on the basis of past dental history, clinical parameters and radiographic patterns of alveolar bone loss. They have been suffering from periodontal disease and have received scaling and root planning. The mean pocket depth of these patients was 6.05mm (Table 1).

Thirty breast cancer patients were diagnosed with invasive ductal carcinoma from UNIMED Medical Institute, Hong Kong. The age of the patients ranged from 27 to 79 (mean age was 52.6). The clinic-pathological characteristics of breast cancer patients were summarized in Table 2. They were free from other systemic diseases and periodontitis. They had also been examined orally at Keenlink Dental Clinic, Hong Kong. These patients received 4 cycles of 500mg/m^2^ 5-fluorouracil, 75 mg/m^2^ epirubicin and 500mg/m^2^ cyclophosphamide at a 3-week interval between each cycle. Informed consents were collected before commencement of this study. The experimental protocol has been approved by the ethics committee of Sichuan University and The University of Hong Kong (Protocol No. FEC-DOC-CXB-AB). Their mean pocket depth was 3.6mm, and clinical attachment loss was less than 1 mm in any one quadrant.

**Table 2:** Clinicopathological characteristics of 30 breast cancer patients

	Total number of patients (N) = 30
**Mean age**	52.6 (range: 27–79)
**Menopausal status**	
Premenopausal	7
Postmenopausal	23
**Histological Nodal Status**	
Negative	26
Positive	4
**Tumor Size**	
≤ 2cm (T1)	15
2–5cm (T2)	14
> 5cm (T3)	1
**Histology**	
Ductal	28
Lobular	2
**Grading**	
G1	16
G2	12
G3	2
**Lymphovascular Permeation**	
Absence	26
Presence	4
**ER Status**	
Negative	21
Positive	9
**PR Status**	
Negative	22
Positive	8
**Her2/neu Status**	
Negative	25
Positive	5

### Cardiac markers measurement

Peripheral venous blood was taken for complete blood picture for all groups’ subjects by a automation blood system (SYSMEX, XS-800i, Japan). The troponin T, troponin I, pro-BNP, and LDH were measured from their serum samples using an automation closed kit system of COBAS INTEGRA 400 PLUS, and ELECSYS 2010 (ROCHE, Germany). The marker levels were obtained by comparing with the standard curve generated from the standards provided by the manufacturer.

### Measurement of high sensitivity C-reactive protein and P-selectin

The High sensitivity C-reactive protein was measured from subjects’ serum samples using an automation closed kit system of COBAS INTEGRA 400 PLUS (Roche, Germany). The marker level was also obtained by comparing with the standard curve generated from the standards provided by the manufacturer.

The P-selectin Enzyme-linked immunosorbent assay (ELISA) kit (Roche, Germany) was used. The serum was separated from the blood, centrifuged at 13000 rotations per minute (rpm) prior to the assay. The samples and control-serum were diluted to the following ration: 1:50. After, 100 μl of standard solution and serum were pipetted into the appropriate well. 50 μl of immunoreagent was added and covered with adhesive cover foil, then incubated and shaken at 300 rpm for 1 hour. The buffer was aspirated away, and the plate was washed 3 times (1 minute each) with washing buffer. The washing buffer was again aspirated away. 100 μl of TMB substrate was added and incubated on a shaker at 300 rpm for 25 minutes at room temperature. 25 μl of TMB stop solution was added to each well after colour development was sufficient. It was then incubated for 1 minute on the shaker, and measured within 5 minutes at 450 nm.

The mean levels of cardiac and inflammatory markers were compared using students’ T-test (SPSS, 15.0, USA). The level of significance was p < 0.05.

## Results

The complete blood picture of various groups is shown in Table 3. All the parameters in healthy controls and CP patients lied within the normal range. In the cancer patient group, the haemoglobin, platelet and lymphocyte levels were below the normal range, but the neutrophil count demonstrated a statistically significant increase (p < 0.05) compared to the other two groups. White blood cell count of the CP group was also significantly higher than the healthy controls (p < 0.05). The rest of the parameters remained similar in both CP and cancer patients.

**Table 3:** Blood picture (Mean±SD) of control and test groups

Blood cells	Healthy controls (N=108)	Periodontitis patients (N=44)	Cancer patients (N=30)	Normal range	Units
White blood cell	6.32(±1.11)	5.75(±1.40)	5.37(±1.49)	4.00-11.00	10^9^/L
Red blood cell	4.47(±0.88)	3.82(±1.06)	4.18(±1.10)	3.8-5.80	10^12^/L
Hemoglobin	12.5(±4.26)	12.3(±2.69)	9.6(±4.89)	11.5-16.5	g/dl
Platelet	319(±86.16)	191(±74.77)	115(±48.52)	150-400	10^9^/L
Neutrophils	66.9(±0.81)	49.8(±6.64)	79.5(±9.07)	43.7-77.1	%
Lymphocytes	25.4(±3.07)	39.6(±1.48)*	11.4(±1.46)	15-45.8	%
Monocyte	5.7(±0.26)	7.4(±0.48)	7.4(±0.94)	4.7-12.2	%
Eosinophil	1.7(±0.45)	2.7(±0.33)	1.5(±0.43)	0.7-7.0	%
Basophil	0.3(±0.04)	0.5(±0.03)	0.2(±0.04)	0.1-1.2	%

Significant different from the control, * *p* < 0.05

The means of all markers were significantly higher in CP and cancer patients (p < 0.01) than the healthy controls (Table 4). Moreover, the level of these markers in the cancer group was higher than CP patients except for Hs-CRP and P-selectin. Although healthy controls demonstrated the lowest level of the markers among the three groups, their LDH and NT pro-BNP levels did not fall in the normal range. In fact, the level of NT pro-BNP in all three groups was lower than the normal range.

**Table 4:** Expression level (Mean ± SE) of inflammatory and cardiac markers

Cardiac & InflammatoryMarkers	Groups	Mean values	Normal Range & Unit
	1	305(±4.644)	
LDH	2	485(±11.272)*	240-480 U/L
	3	678(±33.989)*	
	1	1.03(±0.041)	
Troponin T	2	10.47(±0.407)*	< 10 ng/ml
	3	18.10(±0.910)*	
	1	0.19(±0.007)	
Troponin I	2	1.88(±0.073)*	0-2.0 ng/ml
	3	3.44(±0.173)*	
	1	82.97(±1.040)	
NT pro-BNP	2	115.77(±1.002)*	140-320 pg/ml
	3	148.55(±1.650)*	
	1	0.39(±0.004)	
P-selectin	2	1.23(±0.051)*	0.6-10 ng/ml
	3	0.91(±0.008)*	
High sensitive	1	0.51(±0.030)	
C-reactive	2	2.82(±0.166)*	< 5 mg/L
protein	3	3.35(±0.261)*	

Significant difference from control, * *p* < 0.05

1= Control subjects (N=108); 2= Periodontitis patients (N = 44); 3= Cancer patients (N = 30)

## Discussion

Our results showed that chronic periodontitis (CP) was associated with increased serum levels of LDH, troponins T & I, NT pro-BNP, and P selectin. The underlying mechanisms between CP and CHD are not fully understood, but several pathways have been proposed, such as periodontal infection, which can have both direct and indirect effects.

Bacteria are an important causative agent in periodontitis. Bacteremia occurs when bacteria enter the bloodstream from the oral cavity during tooth brushing, normal mastication, after tooth extraction or periodontal treatment, and inflammatory activity of periodontitis [49]. The bacteria then infects epithelial integrity in periodontal pockets [50], and vascular endothelium of the arterial wall. These can lead to tooth loss and trigger a systemic response and atherosclerosis [51,52]. Chronic infection has been reported to be a contribution to atherosclerosis [53].

Periodontal pathogens are associated with MI and CHD by activating mononuclear phagocytes. When the liposaccharide (products of the pathogens) concentrations are low, the secretions of interleukins 1α and 1β, and tumour necrosis factors increase. These show that cytokines play a role in atherothrombogenesis inflammatory response [54]. The secretion of cytokines also promotes production of lymphocytes [55,56]. The white blood cell count was found to be higher in CP patients in our study which is consistent with Shi’s study [57].

An increased number of white blood cells is associated with an increased risk of CHD, cardiovascular disease, atherosclerosis, thrombosis, and myocardial ischemia [58-60]. This increase is probably caused by the inflammatory nature of chronic infections [61], and also due to the polymophonuclear leukocytes [62]. As a result of the increased leukocytes, more cells adhere to the endothelial cells of the blood vessels. These eventually decrease the blood flow as well as the red blood cell count [63]. In CP patients, red blood cell count was found to be lower than healthy controls [64]. This indicated that anaemia might play a role in CHD.

Neutrophils are produced in bone marrow and are important for detecting and dispose the microorganisms. The neutrophil level is important when planning dental treatment for cancer patients who have received chemotherapy and radiotherapy, as their bone marrow may well be affected [65]. A neutrophil count may indicate any bacterial proliferation.

On the other hand, some studies have found that people who have been infected with *Chlamydia pneumoniae* (*C. pneumonia*) have a higher risk of CHD [66,67]. The *C. pneumoniae* replicates in the macrophages via chewing and tooth brushing; they are then transported to the arteries from the lungs [68,69]. Furthermore, recent studies have shown that *Aggregatibacter actinomycetemcomitans*, *Porphyromonas gingivalis*, and *Prevotella intermedia* are able to invade the coronary endothelium, and the first two were also found in atherosclerotic plaque [70,71].

Our results for the cardiac and inflammatory markers are consistent with the existing literature. A study by Bruno Zappacosta found a statistically significant increase in LDH in the periodontal patient group [72]. The association between periodontitis with serum endotoxin/lipopolysaccharides (LPS) and increased troponins have been found in Goteiner’s study [73]. Papapanagiotou found that a significantly increased plasma level of P-selectin was related to periodontitis [74].

Some researchers found that in healthy populations, the hs-CRP could be measured in a lower concentration (range of 0.1 to 10 mg/L) than 100-1000-fold of the acute phase infection and inflammation patients, and there were 2-fold higher hs-CRP concentrations in patients with stable CHD [75]. These results are overall in line with ours (Table 4).

The hs-CRP cut-off point for high-risk of future development of CHD is likely to be >1.0 mg/L in a general population of Japanese, and this value is much lower than Western populations [76]. Elevated hs-CRP levels were also found periodontits patients [27].

Cardiac markers are useful in diagnosing acute coronary syndromes [77,78]. Although LDH are concentrated in the heart cells, they are found in nearly all body cells and are released into the bloodstream when the cells are damaged. Therefore, they cannot be used to diagnose particular diseases, such as CHD. Troponins have high specificity and sensitivity; thus, they can be used as a gold standard to detect myocardial damage [79].

In the present study, other confounding factors might to some extent affect the results, as both periodontal disease and CHD share many common risk factors, such as smoking, increased consumption of saturated fat and cholesterol, diabetes, blood pressure, and decreased consumption of vegetables and fruits [80,81].

## Conclusions

Within the limitations concerned above, the present study shows that subjects with chronic periodontitis exhibit significantly higher levels of inflammatory and cardiac biomarkers than the controls. These findings suggest that CP increases the systemic levels of inflammation and potential risks for CHD. Hence, periodontal disease may represent a modifiable risk factor for CHD.

## Abbreviations

5FU: 5-fluorouracil; *C. pneumonia: Chlamydia pneumonia*; CP: chronic periodontitis; CHD: coronary heart disease; ELISA: Enzyme-linked immunosorbent assay; hs-CRP: high sensitivity C-reactive protein; IDC: invasive ductal carcinoma; LDH: lactate dehydrogenase; LPS: lipopolysaccharides; MI: myocardial infarction; Nt-proBNP: N-terminal pro brain natriuretic peptide; rpm: rotations per minute.

## Competing interests

We declare that we have no financial and personal relationships with other people or organizations that can inappropriately influence our work. There is no professional or other personal interest of any nature or kind in any product, service and/or company that could be construed as influencing the position presented in the article entitled, “Comparing serum levels of cardiac biomarkers in cancer patients receiving chemotherapy and subjects with chronic periodontitis”.

## Authors' contributions

WTYL conducted the research, performed data collection and data analysis, and participated in manuscript writing. YY, CF, JL and YT conducted the clinical examination and performed data collection. LB performed data collection and data analysis. MW supervised clinical examination and participated in manuscript planning. HL performed data analysis and participated manuscript writing. MNBC conducted the clinical examination and participated in manuscript writing. LWCC participated in manuscript planning and writing. All authors read and approved the final manuscript.
